# Chronic Waterborne Exposure to Polystyrene Microplastics Induces Kupffer Cell Polarization Imbalance and Hepatic Lipid Accumulation

**DOI:** 10.1096/fj.202500910RR

**Published:** 2025-09-03

**Authors:** Ting Li, Zhenghong Yao, Yuxin Huang, Ran Li, Lu Zhang, Rucheng Chen, Weijia Gu

**Affiliations:** ^1^ School of Public Health Zhejiang Chinese Medical University Hangzhou China; ^2^ Zhejiang International Science and Technology Cooperation Base of Air Pollution and Health Hangzhou China

**Keywords:** IL‐17, Kupffer cell, lipid metabolism, polystyrene microplastics

## Abstract

Microplastics (MPs), particles under 5 mm, are widespread environmental contaminants. Polystyrene (PS), used in many household items, degrades into polystyrene MPs (PS‐MPs), which accumulate in the environment. Chronic exposure to waterborne PS‐MPs was found to disrupt hepatic lipid metabolism in C57BL/6N mice through inflammatory Kupffer cell polarization and IL‐17/NF‐κB signaling pathways. While short‐term PS‐MPs exposure revealed preferential accumulation in the liver and testes, long‐term exposure (9–12 weeks) induced significant increases in body fat percentage and hepatic lipid deposition independent of dietary changes. Mechanistically, chronic PS‐MPs exposure promoted Kupffer cell polarization toward pro‐inflammatory M1 phenotypes, accompanied by upregulated IL‐17 expression and suppressed anti‐inflammatory cytokines. Western blot analysis demonstrated concurrent elevation of lipid synthesis markers with reduced lipid oxidation and transport proteins. These findings established that PS‐MPs accumulation drives hepatic steatosis through dual mechanisms of macrophage‐mediated inflammation and impaired lipid homeostasis pathways.

## Graphical abstract

Chronic Polystyrene Microplastic (PS‐MP) Exposure Drives Hepatic Steatosis via Dual Inflammatory and Metabolic Pathways. Chronic PS‐MP exposure in C57BL/6N mice induces hepatic lipid deposition and elevated body fat through M1‐polarized Kupffer cells, IL‐17A/NF‐κB activation, and impaired lipid homeostasis. Mechanistically, PS‐MPs upregulate lipid synthesis while suppressing oxidation and transport proteins, linking environmental MPs to metabolic dysfunction.

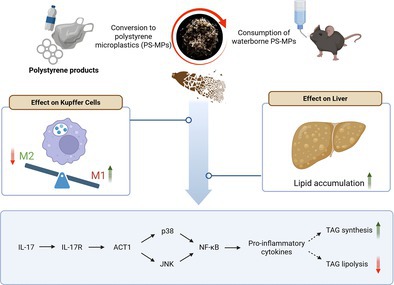

## 1 Introduction

Microplastics (MPs), defined as plastic particles under 5 mm in diameter and composed of polymers with various additives [1], are widespread contaminants detected in drinking water, air, and food sources like seafood, salt, and tea [2, 3, 4, 5]. Polystyrene (PS), commonly used in household items like food containers, toys, and toothbrushes [6, 7], contributes substantially to environmental PS‐MPs accumulation as it degrades [8]. In healthy volunteers, PS‐MPs have been detected in blood at concentrations as high as 4.8 μg/mL [9], with drinking water (both tap and bottled) as the primary exposure source [10, 11, 12, 13]. Patients with cirrhosis show particularly high levels of PS‐MPs, reaching 4.6–11.9 particles per gram of liver tissue, far above levels in healthy individuals [14]. Previous studies have investigated the effects of PS‐MPs with varying particle sizes and exposure durations. For example, exposure to 5 and 20 μm PS‐MPs for 30 days has been employed to examine their distribution, accumulation in liver tissue, and tissue‐specific health risks in mice [15]. Similarly, exposure to 0.5, 4, and 10 μm PS‐MPs for 180 days has been used to evaluate their neurotoxicity and potential effects on the nervous system [16]. These findings highlight the significance of particle size and exposure duration in determining the biological effects and toxicity of PS‐MPs across various organ systems. Given the essential role of the gut‐liver axis in the intake, metabolism, and excretion of MPs—and our observation that the liver is a primary target organ following waterborne PS‐MPs exposure, with PS‐MPs inducing hepatic lipid accumulation [17]—research on the mechanisms underlying MPs‐induced hepatic lipid deposition remains limited.

Kupffer cells (KCs), the resident macrophages of the liver, act as primary phagocytes, effectively clearing particles and serving as a crucial first line of defense against MPs [18, 19]. Research shows that MPs ranging from 5 to 20 μm can accumulate in murine livers, leading to hepatic inflammation [20]. Upon phagocytosis of external particles, KCs release pro‐inflammatory cytokines, including interleukin‐1 (IL‐1), IL‐6, and IL‐17 [21, 22]. Notably, IL‐17 signaling is known to accelerate the progression of nonalcoholic fatty liver disease by regulating lipogenesis, including de novo lipogenesis, lipid β‐oxidation, and lipid secretion [23, 24]. Therefore, the activation of IL‐17 signaling in KCs following the phagocytosis of PS‐MPs may significantly contribute to the hepatic lipid metabolism abnormalities linked to PS‐MPs.

In this study, we established both short‐term and chronic exposure models in mice by administering PS‐MPs via drinking water to investigate the inflammatory response of KCs and its effects on hepatic lipid metabolism. This approach aimed to clarify the mechanisms by which waterborne PS‐MPs exposure disrupts lipid metabolism in the body.

## 2 Materials and Methods

### 2.1 Reagents and Antibodies

The reagents and antibodies used in this study were as follows: Polystyrene microplastics (PS‐MPs, 1 μm, #6‐1‐0100) and red fluorescent PS‐MPs (F‐PS‐MPs, 1 μm, #7‐1‐0100) were obtained from Tianjin Besler Chromatography Technology Development Center (Tianjin, China). Other reagents included DMEM (#8123594, Gibco, China), collagenase (#LS004188, Worthington Biochemical Corporation, USA), Deoxyribonuclease I (#LS002138, Worthington Biochemical Corporation, USA), Percoll (#17089109‐1, Cytiva, China), red blood cell lysis buffer (RT12202, TIANGEN, China), PBS (#24143B37, Biosharp, China), 7‐amino‐actinomycin D (7‐AAD) (#559925, BD Pharmingen, USA), Fc blocking (#553141, BD Pharmingen, USA), RNAiso Plus reagent (#9109, TaKaRa, Japan), PrimeScript RT Master Mix (RR036B, TaKaRa, Japan), PowerUP SYBR Green Master Mix (A25742, Applied Biosystems, USA), RIPA lysis buffer (AR0105‐100, Boster Biological Technology, USA), BCA protein assay kit (P0010, Beyotime Biotechnology, China), Enzymatic determination kit for triglyceride (TG) content in liver and fat samples (E1025‐105, Applygen Technologies Inc., China), Enzymatic determination kit for total cholesterol (TC) content in liver and fat samples (E1026‐105, Applygen Technologies Inc., China), Free fatty acid (FFA) content determination kit (E1001, Applygen Technologies Inc., China), and Fatty Acid Synthase (FASN) Activity Assay Kit (BC0550, Solarbio life sciences, China).

The antibodies used were as follows: FITC Rat Anti‐CD11b (M1/70, #557396, BD Pharmingen, USA), BV421 Rat Anti‐Mouse F4/80 (T45‐2342, #565411, BD Pharmingen, USA), APC‐Cy7 Rat Anti‐Mouse CD45R (RA3‐6B2, #552094, BD Pharmingen, USA), PE Rat Anti‐Mouse CD86 (GL1, #553692, BD Pharmingen, USA), Alexa Fluor 647 Rat Anti‐Mouse CD206 (MR5D3, #565250, BD Pharmingen, USA), Interleukin 17 polyclonal antibody (IL17, #66148‐1‐IG, Proteintech, USA), IL‐17RA Polyclonal antibody (IL17RA, #32055‐1‐AP, Proteintech, USA), p38 MAPK polyclonal antibody (p38, #14064‐1‐AP, Proteintech, USA), Phospho‐p38 (Thr180/Tyr182) (p‐p38, #4511, CST, USA), JNK polyclonal antibody (JNK, #51151‐1‐AP, Proteintech, USA), Phospho‐JNK (Thr183/Tyr185) (p‐JNK, #4668, CST, USA), Erk1/2 (ERK, #4695, CST, USA), Phospho‐ERK (Thr202/Tyr204) (p‐ERK, #4370, CST, USA), IkB alpha polyclonal antibody (IκBα, #10268‐1‐AP, Proteintech, USA), p‐NFKBIA/IkB alpha Antibody (B‐9) (p‐IκBα, sc‐8404, Santa Cruz, USA), NF‐κB p65 Polyclonal antibody (NF‐κB, #10745‐1‐AP, Proteintech, USA), Phospho‐NF‐κB p65 (Ser536) (93H1) (p‐NF‐κB^p65^, #3033S, CST, USA), ACC1 Polyclonal antibody (Acetyl CoA Carboxylase 1, #21923‐1‐AP, Proteintech, USA), FASN Polyclonal antibody (FASN, #10624‐2‐AP, Proteintech, USA), Phospho‐ACC1 (Ser79) Polyclonal antibody (p‐ACC1^Ser79^, #29119‐1‐AP, Proteintech, USA), SREBP‐1 Antibody (2A4) (Sterol‐regulatory Element Binding Protein 1, sc‐13551, Santa Cruz, USA), CPT1α Polyclonal antibody (Carnitine Palmitoyltransferase 1α, #15184‐1‐AP, Proteintech, USA), ACOX1 Polyclonal antibody (acyl‐CoA oxidase‐1, #10957‐1‐AP, Proteintech, USA), ATGL Polyclonal antibody (Adipose Triacylglyceride Lipase, #55190‐1‐AP, Proteintech, USA), ApoB Rabbit pAb (Apolipoprotein B, A1330, Abclonal, USA), and GAPDH (14C10) Rabbit mAb (Glyceraldehyde 3‐phosphate dehydrogenase, #2118, CST, USA).

### 2.2 Characterization of PS‐MPs

The morphology of PS‐MPs spheres in aqueous solution was examined using scanning electron microscopy (SEM, JSM‐IT800, JEOL Ltd., Japan). Polydimethylsiloxane (PDLC) films (Chiefway LS Optronics, China) were cut into 2 cm × 2 cm squares and placed in a glass Petri dish containing cyclohexane. The dish was covered with aluminum foil and immersed for 1–3 days to fully separate liquid crystal molecules while maintaining the integrity of the PS‐MPs' spherical structure. After immersion, the films were dried in a drying oven for 4–8 h to evaporate the cyclohexane, leaving behind the PS‐MPs spheres. The films were cut into 1 cm × 1 cm pieces and coated with gold for 90 to 150 s for SEM observation.

For size distribution analysis, each sample was dispersed in deionized distilled water at a mass fraction of 0.1%, ultrasonicated for 3 min, and the pH was adjusted. The average particle size distribution and zeta potential were measured using a nanoparticle size analyzer (ZS90, Malvern Instruments, UK), with values over three measurements.

### 2.3 Animals, Animal Care, and PS‐MPs Treatment

Eight‐week‐old male C57BL/6N mice (Charles River Laboratories, Beijing, China) were utilized as experimental animal models. All mice were housed in a temperature‐controlled environment (22°C ± 2°C) with a 12‐h light/12‐h dark cycle and were provided with ad libitum access to food and water. Prior to the start of the experiment, all mice underwent a two‐week acclimatization. All animal experiments were approved by the Animal Care and Use Committee of Zhejiang Chinese Medical University (animal use grant NO. 202306‐0210). After the acclimation period, the mice underwent PS‐MPs exposure in two phases: a short‐term biodistribution study and a long‐term exposure experiment.

For the short‐term biodistribution study, mice were randomly assigned to either the vehicle control (Veh) group or the red fluorescent PS‐MPs (F‐PS) group. The F‐PS group received an oral gavage of 0.2 mg of F‐PS‐MPs [25], while the Veh group was given an equivalent volume of deionized distilled water. In vivo fluorescence imaging of the mice was performed at certain time points. After in vivo fluorescence imaging, the mice were sacrificed. The major organs, including the brain, lungs, liver, kidneys, testes, and adipose tissues (brown adipose tissue [BAT], visceral adipose tissue [VAT], and inguinal adipose tissue [ING]), were harvested, and ex vivo fluorescent images of these organs were obtained under identical conditions.

In the long‐term exposure study, mice were randomly divided into three groups: the vehicle control group (Veh), the low‐dose group (PS‐L, 1 mg/L), and the high‐dose group (PS‐H, 5 mg/L) [3, 26]. PS‐MPs were administered via drinking water during the long‐term exposure. PS‐MPs‐infused water was replaced daily to ensure consistent and stable exposure. Body weight, food intake, and water consumption were measured biweekly, with whole‐body composition analysis at weeks 3, 6, 9, and 12. At the end of week 12, mice were euthanized, and liver tissues were rapidly collected. The left lateral lobe was embedded in optimal cutting temperature (OCT) compound for Oil Red O staining, while the remaining tissue was snap‐frozen in liquid nitrogen and stored at −80°C for subsequent molecular analyses.

### 2.4 In Vivo Fluorescence Imaging

The F‐PS group received an oral gavage of 0.2 mg of F‐PS‐MPs [25], while the Veh group received an equivalent volume of deionized distilled water. Mice were anesthetized with 1.5% isoflurane/oxygen, and abdominal hair was shaved prior to imaging. While imaging, anesthesia was maintained with 1% isoflurane/oxygen delivered through a nose cone system. Mice were positioned ventrally in the in vivo imaging system chamber (IVIS, Lumina XR, Caliper Life Sciences, USA), and pre‐ and post‐injection fluorescence images were taken at 0 min, 30 min, 1 h, 2 h, 4 h, 8 h, and 24 h. Imaging parameters were set as follows: exposure time set to auto, binning at 8, f/stop at 2, and FOV at 13.2 cm. The excitation (EX) filter was set to 605 nm, and the emission (EM) filter was set to 680 nm. Acquired images were processed and analyzed by using Living Imaging software (Version 4.7.4, PerkinElmer, USA). At 24 h post‐administration, mice were sacrificed, and major organs, including the brain, lungs, liver, kidneys, testes, and adipose tissues, were collected. Ex vivo fluorescent imaging of organs was performed under identical conditions using the same system.

### 2.5 Whole‐Body Composition Analysis

At weeks 3, 6, 9, and 12, whole‐body composition analysis was conducted with the mice awake and placed in an acrylic cylinder. A Bruker TD‐NMR system (minispec LF50, Bruker, Billerica, MA, USA) was used to assess body composition, set at a magnetic field of 0.17 T and a pulse frequency of 7.5 MHz. Lean mass, fat mass, and fluid content measurements were recorded, and the mice were returned to their home cages within 1 min. Data were normalized to body weight for analysis [27].

### 2.6 Liver Oil Red Staining

The left lateral lobe was harvested for Oil Red O staining and immediately embedded in OCT compound. The embedded tissue was sectioned at a thickness of 5 μm and subsequently stained with Oil Red O. Sections were immersed in freshly prepared Oil Red O working solution for 8 to 10 min under light‐avoidance conditions. After staining, the sections were briefly air‐dried for 3 s and then sequentially immersed in two baths of 60% isopropanol for 3 and 5 s, respectively, for differentiation. The sections were rinsed in two containers of distilled water for 10 s each. Hematoxylin counterstaining was then performed, followed by microscopic examination and sealing of the sections. For each sample, 4 to 5random sections were analyzed to quantify lipid accumulation. The percentage of Oil Red O‐positive areas in the liver sections was quantitatively measured using ImageJ software (Version 1.54 k, NIH, USA).

### 2.7 Biochemical Analyses of Hepatic TG, TC, and FFA

Hepatic lipids were extracted and quantified to assess TG, TC, and FFA levels. Briefly, approximately 100 mg of liver tissue was homogenized in the appropriate lysis buffer provided with each assay kit, and the homogenate was incubated for 10 min at room temperature to ensure complete lysis. The mixture was then centrifuged, and the resulting supernatant was collected for analysis. TG, TC, and FFA concentrations were determined using commercial assay kits following the manufacturer's instructions. Absorbance was measured using a microplate reader (SpectraMax M3, Molecular Devices, USA) and lipid levels were normalized to either tissue weight or total protein content, as appropriate.

### 2.8 Flow Cytometry for Liver Tissue

Approximately 2 mg of fresh liver tissue was weighed and minced in a petri dish. The minced tissue was transferred to a 50 mL centrifuge tube containing 5 mL of 2% DMEM medium and placed on ice. After all samples were minced, 50 μL of 100 × collagenase and 5 μL of 1000 × DNAzyme were added, and the mixture was digested for 60 min at 37°C. The digested tissues were filtered through a 70 μm cell strainer and triturated with a 1 mL syringe, followed by washing with 10 mL of 2% DMEM medium. After centrifugation at 500 g for 5 min, the supernatant was discarded. The pellet was resuspended in 5 mL of 36% Percoll, transferred to a 15 mL tube, and centrifuged at 500 g for 5 min. The top hepatocyte layer and Percoll middle layer were removed, leaving the pellet. Cell pellets were lysed with 1 mL of 1 × red blood cell lysis buffer for 2 min, after which 5 mL of 2% DMEM medium was added to terminate the reaction. The mixture was then centrifuged at 500 g for 5 min. The cells were washed with 1 × PBS, centrifuged, and resuspended in 2 mL of 1 × PBS. A viability dye, 7‐AAD (1:500), was added, and the cells were incubated for 15 min at room temperature, shielded from light. Cells were washed with 1 mL of 1 × PBS with 2% FBS, centrifuged at 600 g for 3 min, and resuspended in 100 μL of 1 × PBS with 2% FBS. Fc blocking was performed for 15 min, followed by the addition of 2 μL of surface antibodies and incubation for another 15 min. After washing, intracellular staining was performed, followed by centrifugation at 600 g for 3 min. Finally, cells were resuspended in 300 μL of 1 × PBS for flow cytometry analysis on the CytoFLEXS Flow Cytometer (Beckman Coulter, USA). Data were analyzed using Kaluza (Version 2.2.1.20183, Beckman Coulter, USA).

### 2.9 RNA Extraction and Quantitative RT‐PCR Analysis

Total liver RNA was extracted using a TissueLyser‐24 multi‐sample tissue grinder (Jingxin, Shanghai, China) in combination with RNAiso Plus reagent. cDNA was synthesized via reverse transcription using PrimeScript RT Master Mix. Quantitative reverse transcription PCR (qRT‐PCR) was performed on a QuantStudio 7 Flex (Applied Biosystems, USA) with PowerUP SYBR Green Master Mix. The qRT‐PCR protocol comprised the following steps: (1) hold stage (50°C for 2 min and 95°C for 2 min), (2) PCR stage (40 cycles of 95°C for 15 s and 60°C for 1 min), and (3) melt curve stage (95°C for 15 s, 60°C for 1 min, and 95°C for 15 s). Gene expression levels were calculated using the 2^−ΔΔCt^ method and normalized to *Gapdh* expression. The primer sequences for qRT‐PCR are listed in Table 1.

**TABLE 1:** Primers design for real‐time PCR.

Gene	Forward (5′‐3′)	Reverse (5′‐3′)
*Acaca*	AAGGCTATGTGAAGGATG	CTGTCTGAAGAGGTTAGG
*Dgat1*	GAGGACGAGGTGCGAGAC	CAGACGATGGCACCTCAGAT
*Fasn*	GCGGGTTCGTGAAACTGATAA	GCAAAATGGGCCTCCTTGATA
*Il10*	GCTGTCATCGATTTCTCCCCT	GACACCTTGGTCTTGGAGCTTAT
*Il13*	ATGGCCTCTGTAACCGCAAG	CTCATTAGAAGGGGCCGTGG
*Il17a*	ATCTGTGTCTCTGATGCTGTTGCT	CGTGGAACGGTTGAGGTAGTCT
*Il4*	CTCACAGCAACGAAGAACACC	CTGCAGCTCCATGAGAACACT
*Pparg*	ACGTTCTGACAGGACTGTGT	CTGTGTCAACCATGGTAATTTCAGT
*Scd1*	TTCTTGCGATACACTCTGGTGC	CGGGATTGAATGTTCTTGTCGT
*Srebp1*	GTGAGGCGGCTCTGGAACAGAC	ATAGGGGGCGTCAAACAGGCC
*Tgfb1*	AGACCACATCAGCATTGAGTG	GGTGGCAACGAATGTAGCTGT
*Tnfa*	GGAACACGTCGTGGGATAATG	GGCAGACTTTGGATGCTTCTT
*Traf3ip2*	AGCCTGCTCCACCACTTAC	GCTTCGGTTCATTCTAGTTTCTAA
*Gapdh*	AGGTCGGTGTGAACGGATTTG	GGGGTCGTTGATGGCAACA

### 2.10 Protein Extraction and Western Blotting

Following the treatment of liver tissues with RIPA lysis buffer, total protein concentration was determined using a BCA protein assay kit. Protein samples of equal concentration were separated by SDS‐PAGE and transferred to PVDF membranes. The membranes were blocked with 5% non‐fat milk or BSA and incubated with primary antibodies overnight at 4°C. After washing off unbound primary antibodies, secondary antibodies were applied. Target proteins were visualized using a ChemiDoc Touch Imaging System (Bio‐Rad, USA) and quantified using ImageJ software (Version 1.54 K, NIH, USA). GAPDH was used as an internal control to normalize protein expression levels.

### 2.11 Determination of Hepatic FASN Activity

Hepatic FASN activity was measured using a commercial FASN Activity Assay Kit, following the 96‐well plate protocol provided by the manufacturer. Briefly, approximately 100 mg of liver tissue was homogenized in 1 mL of Extraction Reagent I on ice (tissue weight to buffer volume ratio 1:10, w/v). The homogenate was centrifuged at 16000 g for 40 min at 4°C, and the resulting supernatant was collected for enzymatic analysis. The reaction mixture in each well of a 96‐well plate contained 20 μL of the liver tissue supernatant, 4 μL of Reagent II, 4 μL of Reagent III, 164 μL of pre‐warmed Reagent IV (incubated at 40°C for ≥30 min), and 8 μL of Reagent V. A blank well was prepared in parallel by replacing the sample supernatant with 20 μL of distilled water. All components were mixed thoroughly, and the absorbance at 340 nm was measured at 30 s (A1) and 90 s (A2) using a microplate reader (SpectraMax M3, Molecular Devices, USA). The change in absorbance (ΔA = A1 − A2) was used to calculate FASN activity. FASN activity was expressed as units per mg of protein (U/mg protein), where one unit (U) is defined as the amount of enzyme that catalyzes the oxidation of 1 μmol of NADPH per min at 37°C. The activity was calculated according to the following formula:

$$\text{FASN}\;\left(U/\mathrm{mg}\;\text{protein}\right)=\left[\left(\mathrm{\Delta A}\;\text{sample}-\mathrm{\Delta A}\;\text{blank}\right)÷\varepsilon ÷d\times V\;\text{total}\times 10^{6}\right]÷\left(\mathrm{Cpr}\times V\;\text{sample}\right)÷T=3.22\times \left(\mathrm{\Delta A}\;\text{sample}-\mathrm{\Delta A}\;\text{blank}\right)÷\mathrm{Cpr}$$

In this formula, ΔA sample and ΔA blank represent the change in absorbance at 340 nm between 30 and 90 s in the sample and blank wells, respectively. Cpr denotes the protein concentration of the supernatant (mg/mL). The constant 3.22 is derived from the following parameters: the molar extinction coefficient of NADPH (ε = 6.22 × 10^3^ L/mol/cm), the optical path length of the 96‐well plate (d = 0.5 cm), the total reaction volume (V total = 200 μL), the volume of supernatant added to the reaction (V sample = 20 μL), and the reaction time (T = 1 min). Approximately 0.1 g of liver tissue was used for homogenization. All measurements were conducted at 37°C using a microplate reader (SpectraMax M3, Molecular Devices, USA), and protein concentration was quantified using a BCA assay.

### 2.12 Statistical Analysis

All data were presented as mean ± standard deviation (SD) and statistically analyzed using Prism software (version 10.1.2, GraphPad Software, USA). The normality of data distribution was assessed using the Shapiro–Wilk test, and homogeneity of variance was confirmed using Levene's test. Based on these assessments, we determined that our data followed a normal distribution with equal variance, justifying the use of one‐way ANOVA (analysis of variance) followed by Tukey's multiple comparisons test to assess inter‐group interactions and identify treatment effects. In cases where normality or homogeneity assumptions were not met, appropriate non‐parametric tests were applied. The significance level was set at a P value of less than 0.05.

## 3 Results

### 3.1 Characterization of PS‐MPs

**FIGURE 1:**
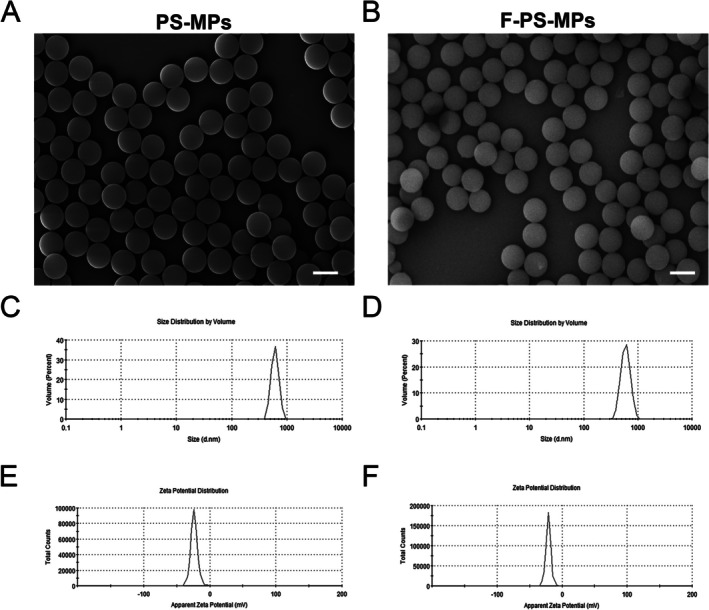
Characterization of PS‐MPs as detected by SEM and nanoparticle size analyzer. (A, B) Representative SEM images of PS‐MPs (A) and F‐PS‐MPs (B), showing smooth, spherical surfaces. Scale bar = 1 μm. (C, D) Particle size distribution and percentage composition of PS‐MPs (C) and F‐PS‐MPs (D) were measured using a nanoparticle size analyzer. (E, F) Zeta potential distribution of PS‐MPs (E) and F‐PS‐MPs (F).

The morphology of PS‐MPs and F‐PS‐MPs in aqueous solution was examined by SEM. It was demonstrated that both types of MPs microspheres possessed smooth, spherical surfaces (Figure 1A,B). The average particle size distribution and zeta potential were determined using a nanoparticle size analyzer. The particle size of PS‐MPs was 1.036 ± 0.136 μm (Figure 1C), with a zeta potential of −25.53 ± 0.414 mV (Figure 1E). In comparison, the F‐PS‐MPs had a particle size of 0.871 ± 0.069 μm (Figure 1D) and a zeta potential of −22.43 ± 1.159 mV (Figure 1F).

### 3.2 Biodistribution of PS‐MPs in Mice After Short‐Term Exposure

**FIGURE 2:**
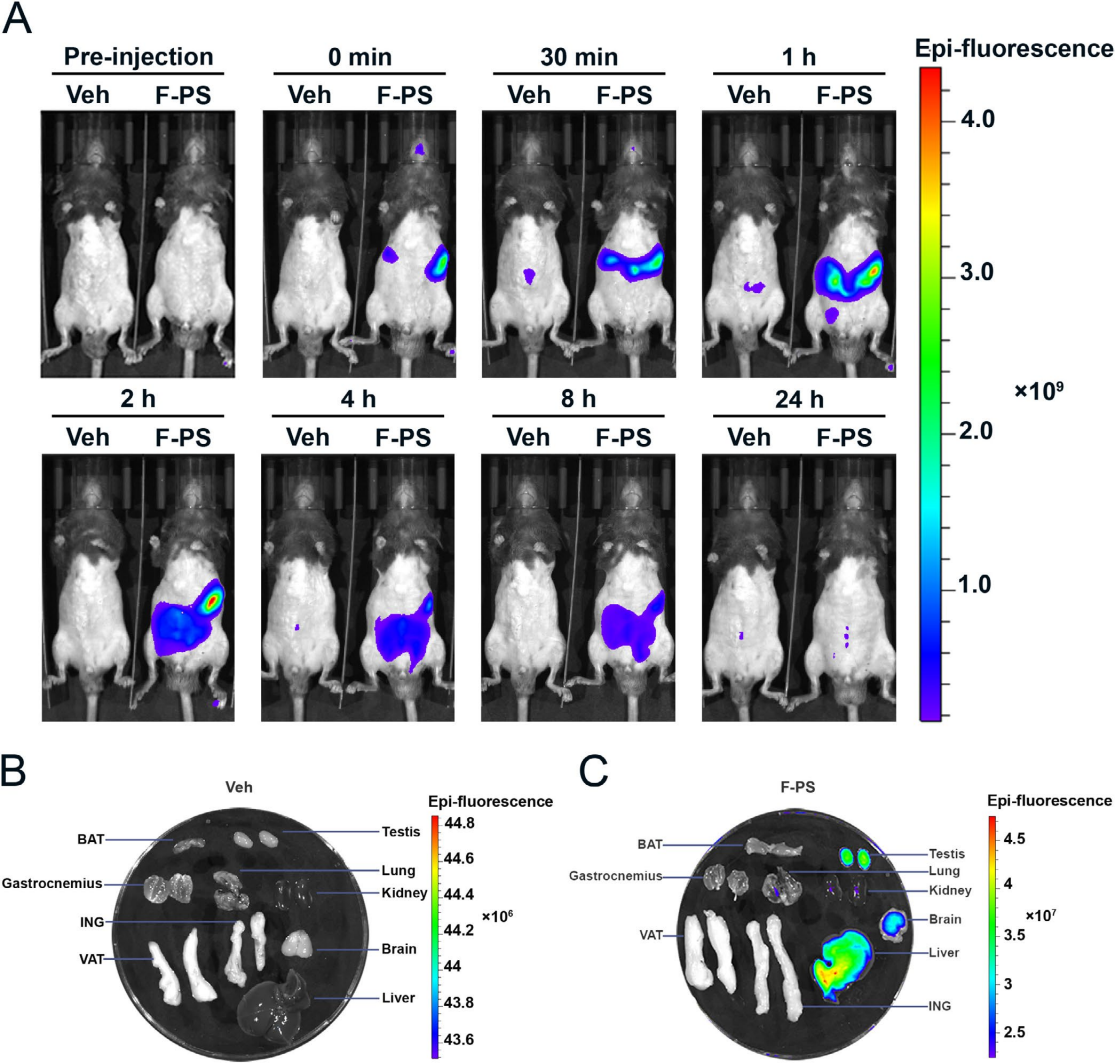
In vivo and ex vivo fluorescence imaging in Veh and F‐PS‐MPs mice, pre‐ and post‐administration of F‐PS‐MPs. (A) Time‐dependent in vivo whole‐body fluorescence images of Veh and F‐PS‐MPs mice captured at 0 min, 30 min, 1 h, 2 h, 4 h, 8 h, and 24 h. (B, C) Ex vivo fluorescence images of organs harvested from Veh and F‐PS‐MPs mice 24 h after F‐PS‐MPs administration, showing PS‐MPs distribution across major organs.

To investigate the biodistribution of PS‐MPs, we conducted a short‐term exposure study in mice using F‐PS‐MPs. Mice were administered 0.2 mg of F‐PS‐MPs via oral gavage [25], followed by in vivo fluorescence imaging conducted pre‐administration and at 0 min, 30 min, 1 h, 2 h, 4 h, 8 h, and 24 h post‐administration. As shown in Figure 2A, PS‐MPs primarily accumulated in the gastrointestinal tract and were largely cleared from the tract within 24 h post‐administration. After completing the in vivo fluorescence imaging, the mice were euthanized, and major organs, including the brain, lungs, liver, kidneys, testes, and adipose tissues (brown adipose tissue [BAT], visceral adipose tissue [VAT], and inguinal adipose tissue [ING]) were harvested. Ex vivo fluorescent images of the organs were captured, revealing that the fluorescence intensity in the liver and testes of F‐PS‐MPs treated mice was significantly higher than in other organs, suggesting that the liver and testes may act as target organs for PS‐MPs.

### 3.3 Chronic Exposure to PS‐MPs Increased Body Fat Percentage and Induced Lipid Accumulation in the Liver

**FIGURE 3:**
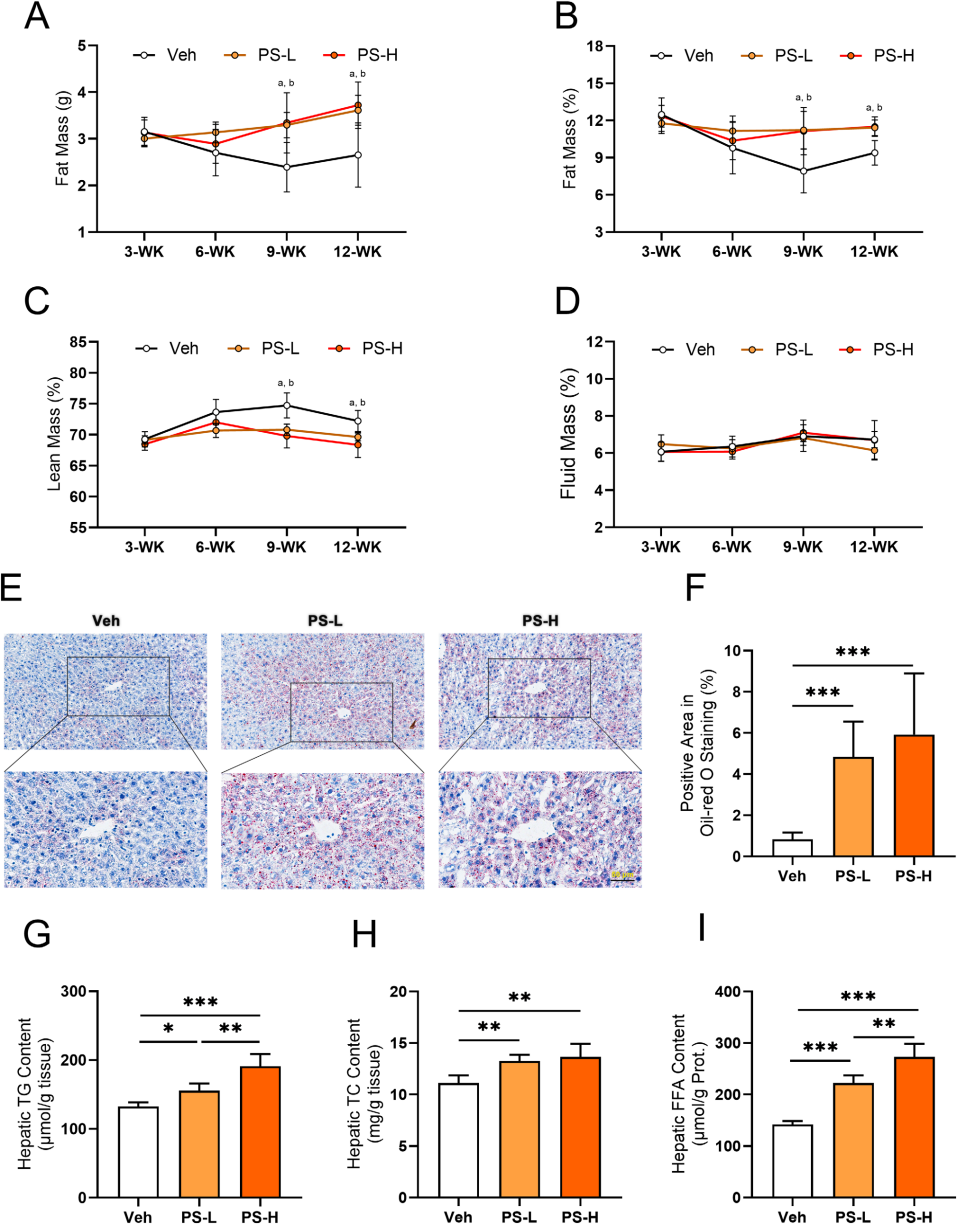
Metabolic characteristics of mice with 12‐week exposure to PS‐MPs in drinking water. (A–D) The absolute fat weight (A), fat mass percentage (B), lean mass percentage (C), and free body fluid percentage (D) at various time points during the experiment for Veh, PS‐L, and PS‐H mice. (E) Representative images of Oil‐red O staining in liver sections. Scale bar = 50 μm. (F) Quantification of Oil‐red O positive area as a percentage of total area. (G) Total TG levels in the hepatic lipid extracts. (H) Total TC levels in the hepatic lipid extracts. (I) Total FFA levels in the hepatic lipid extracts. Values are expressed as mean ± SD, *n* = 6–7; ^a^PS‐L vs. Veh, *p* < 0.05; ^b^PS‐H vs. Veh, *p* < 0.05; ***p < 0.001.

To further elucidate the effects of chronic exposure to PS‐MPs on the body, we conducted a 12‐week exposure to PS‐MPs via drinking water with adult male mice. The mice were divided into the PS‐L group (1 mg/L) and the PS‐H group (5 mg/L), simulating human daily exposure to PS‐MPs through the digestive tract [3, 26]. After 12 weeks of continuous exposure to PS‐MPs through drinking water, no significant differences in body weight, food intake, or water intake were observed among the groups (Figure S1A–C). However, body composition analysis showed a significant increase in both the absolute fat weight and the percentage of fat mass relative to body weight in the PS‐L and PS‐H groups compared to the Veh group, beginning at week 9 (Figure 3A,B). Conversely, the percentage of lean mass relative to body weight significantly decreased from week 9 onward (Figure 3C). Throughout the duration of the experiment, no notable changes in body fluid content were observed among the groups (Figure 3D).

In the foregoing experiment, we identified the liver, a critical organ of lipid metabolism, as the primary site of PS‐MPs accumulation. Consequently, Oil Red O staining was conducted to assess hepatic lipid deposition. The results revealed significant lipid accumulation in both the PS‐L and PS‐H groups compared to the Veh group (Figure 3E,F, Figure S2). The levels of hepatic TG, TC, and FFA revealed significant and dose‐dependent increases in PS‐MPs‐exposed groups, consistent with the histopathological findings (Figure 3G–I).

### 3.4 Chronic Exposure to PS‐MPs Induced M1 Polarization of Hepatic KCs in the Liver via IL‐17RC/ACT1/MAPK Signaling

**FIGURE 4:**
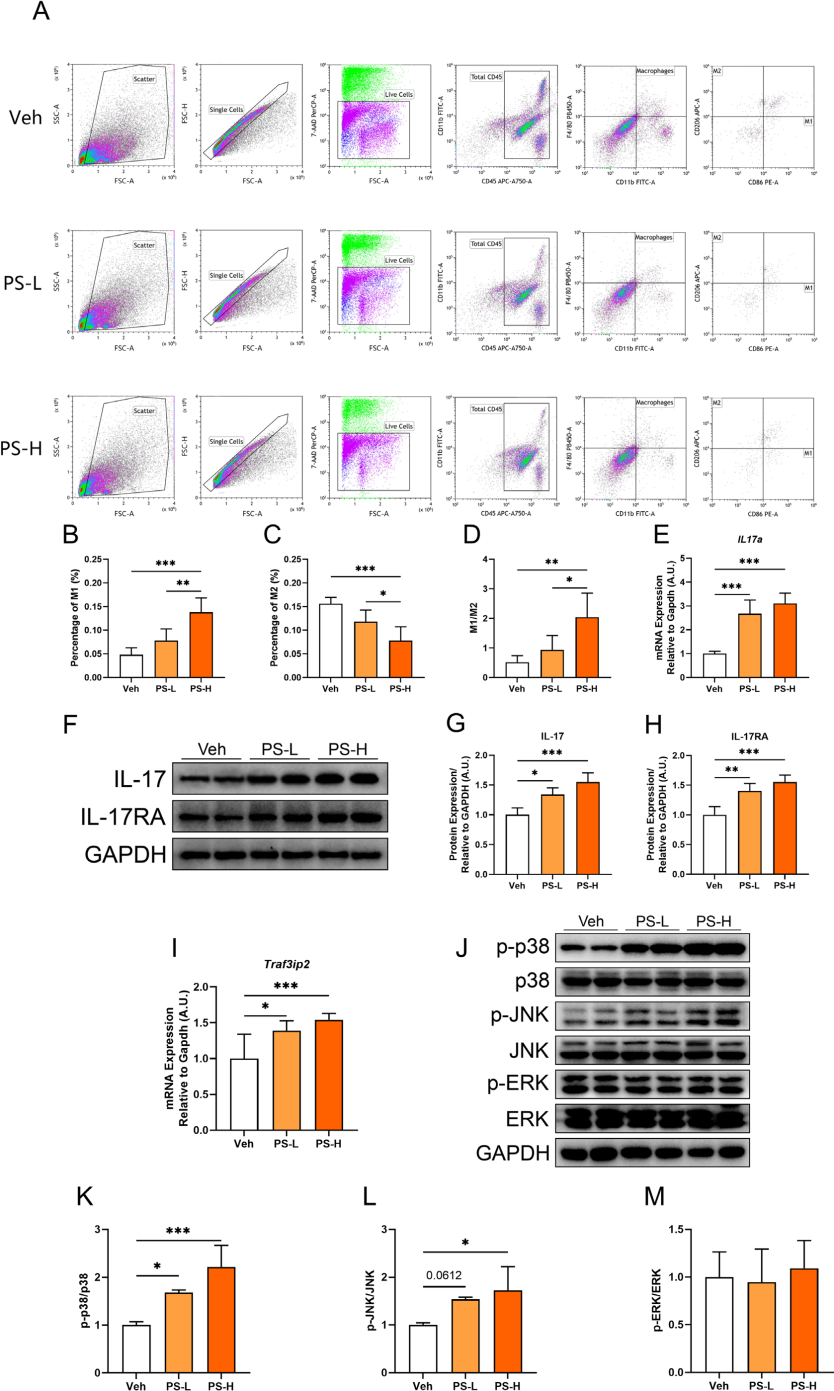
Analysis of the polarization of KCs in mouse liver after chronic exposure to PS‐MPs via flow cytometry. (A) Representative flow cytometry plots showing the gating strategy used to measure the frequency of liver F4/80^+^CD11b^+^CD86^+^CD206^−^ (M1) or F4/80^+^CD11b^+^CD86^−^CD206^+^ (M2) KCs. Percentage of M1 KCs (B), precentage of M2 KCs (C), and M1/M2 ratio in the liver (D). (E) The mRNA levels of *Il17a*. (F–H) Representative protein bands (F) and analysis of IL‐17 (G) and IL‐17RA (H). (I) The mRNA levels of *Traf3ip2*. (J–M) Representative protein bands (J) and analysis of p‐p38 (K), p‐JNK (L), and p‐ERK (M). Values are expressed as mean ± SD, *n* = 6–7; *p < 0.05; **p < 0.01; ***p < 0.001.

KCs, the resident macrophages of the liver, function as primary phagocytes that effectively clear PS‐MPs. To investigate the impact of PS‐MPs on KCs, we performed flow cytometry analysis on liver tissue. As shown in Figure 4A–C, compared to the Veh and PS‐L groups, the percentage of M1‐type KCs among macrophages significantly increased in the PS‐H group, while the percentage of M2‐type KCs markedly decreased. This finding is corroborated by the observed M1/M2 ratio in the liver (Figure 4D).

Based on the observation that PS‐MPs induced the polarization of hepatic KCs toward the M1 phenotype, we further analyzed cytokine expression levels in the liver. A significant increase in the pro‐inflammatory cytokine *Il17a* was detected in both the PS‐L and PS‐H groups compared to the Veh group (Figure 4E). Western blot analysis was subsequently performed to assess the protein levels of IL‐17 and its downstream receptor IL‐17RA, revealing a dose‐dependent increase in both proteins following PS‐MPs exposure (Figure 4F–H). Additionally, the expression of *Traf3ip2*, the gene encoding the IL‐17 signaling adaptor ACT1, was significantly upregulated in the PS‐L and PS‐H groups compared to the Veh group (Figure 4I). Further investigation of downstream components of the IL‐17 signaling pathway demonstrated a significant increase in the phosphorylation levels of p38 and JNK MAPKs in the PS‐L and PS‐H groups compared to the Veh group (Figure 4J–L). However, no significant changes in ERK phosphorylation levels were observed following PS‐MPs exposure (Figure 4J,M). These results suggested that PS‐MPs may disrupt the balance of KCs polarization, potentially leading to liver inflammation.

### 3.5 Chronic Exposure to PS‐MPs Activated Hepatic NF‐κB Signaling and Inhibited Anti‐Inflammatory Cytokine Production

**FIGURE 5:**
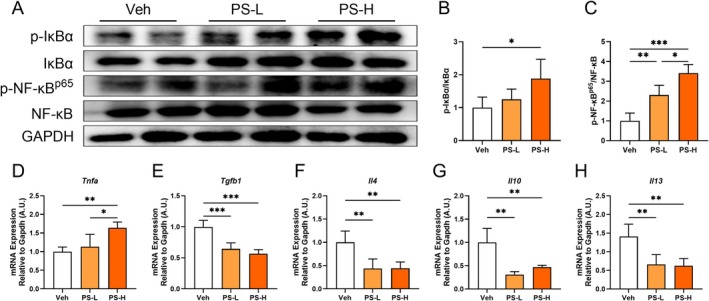
Chronic exposure to PS‐MPs activated hepatic NF‐κB signaling and inhibited anti‐inflammatory cytokine production. (A–C) Representative protein bands and analysis of p‐IκBα (B) and p‐NF‐κB (C) across groups. (D–H) The mRNA levels of *Tnfa* (D), *Tgfb1* (E), *Il4* (F), *Il10* (G), and *Il13* (H) in liver tissues from the Veh, PS‐L, and PS‐H groups. Values are expressed as mean ± SD, *n* = 6–7; *p < 0.05; **p < 0.01; ***p < 0.001.

The NF‐κB signaling pathway plays a crucial role in regulating inflammation in the liver. Following the confirmation of KCs polarization toward the pro‐inflammatory M1 phenotype, we further analyzed changes in NF‐κB activation and downstream inflammatory cytokines. The results demonstrated a significant elevation in the levels of p‐IκBα, a key regulator of inflammatory cytokine expression, in the PS‐H group compared to the Veh group (Figure 5A,B). Moreover, p‐NF‐κB^p65^ levels increased in a dose‐dependent manner with PS‐MPs exposure (Figure 5A,C).

Additionally, the pro‐inflammatory cytokine *Tnfa* was significantly upregulated in the PS‐H group compared to the Veh group (Figure 5D). In contrast, the expression of anti‐inflammatory cytokines, including *Tgfb1*, *Il4*, *Il10*, and *Il13*, was significantly reduced in both the PS‐L and PS‐H groups compared to the Veh group (Figure 5E–H).

Collectively, these findings suggested that PS‐MPs exposure activated hepatic NF‐κB signaling while concurrently suppressing the production of anti‐inflammatory cytokines.

### 3.6 PS‐MPs Enhanced Hepatic Lipid Synthesis While Inhibiting Lipid Oxidation and Transport

**FIGURE 6:**
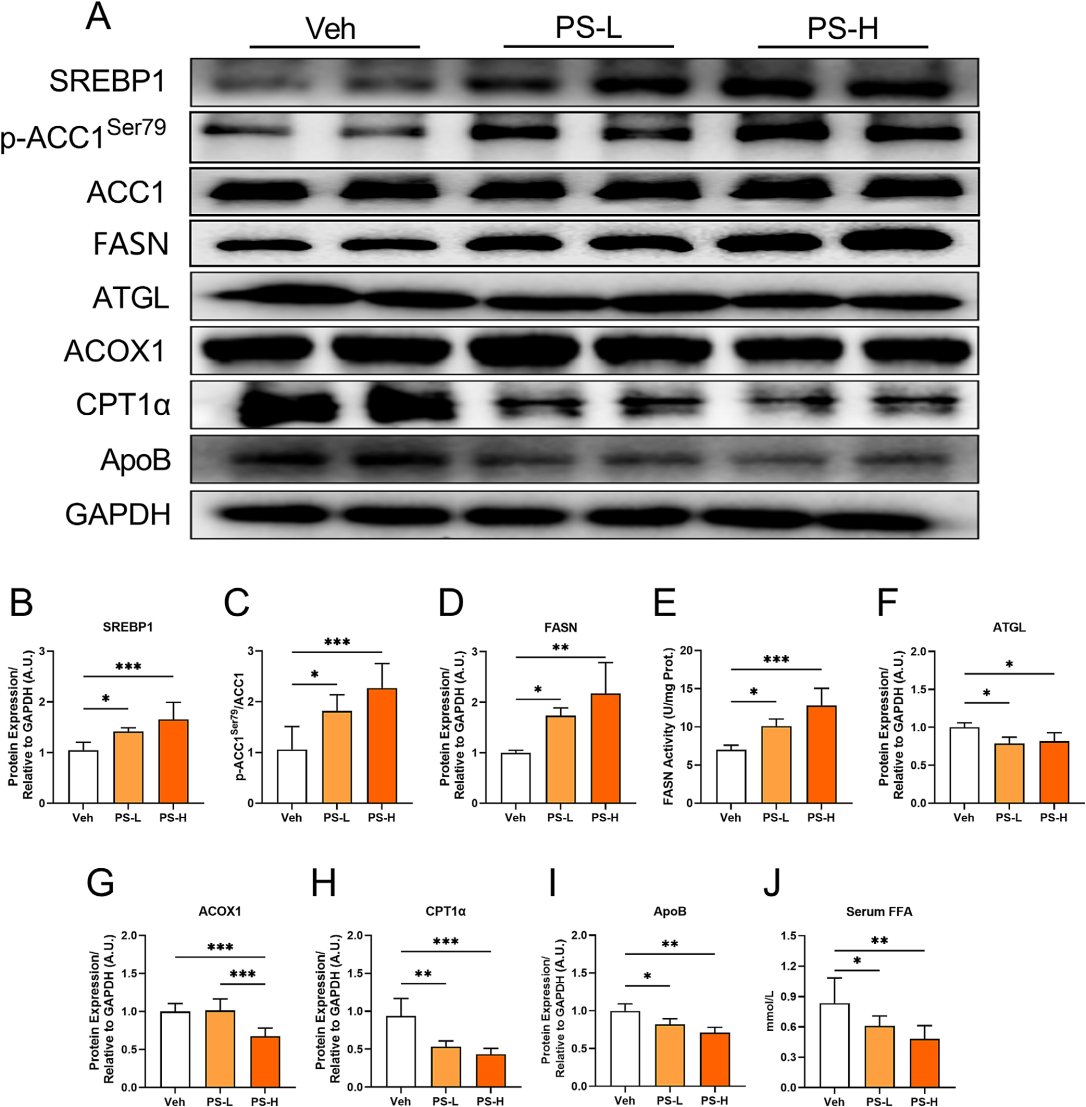
Analysis of key proteins involved in lipid metabolism in the livers of mice exposed to chronic PS‐MPs via western blot. (A–G) Representative protein bands (A) and the analysis of SREBP1 (B), p‐ACC1^Ser79^ (C), FASN (D), ATGL (F), ACOX1 (G), CPT1α (H), and ApoB (I) in liver tissues from the Veh, PS‐L, and PS‐H groups. (E) The activity of FASN in liver tissues from the Veh, PS‐L, and PS‐H groups. (J) The levels of FFA in serum from the Veh, PS‐L, and PS‐H groups. Values are expressed as mean ± SD, *n* = 6–7; *p < 0.05; **p < 0.01; ***p < 0.001.

Research indicates that increased activation of the interleukin IL‐17 axis regulates liver damage, and neutralizing IL‐17A alleviates obesity‐driven hepatocellular damage in mice [23]. We further explored whether PS‐MPs‐driven IL‐17 influenced the development and progression of hepatic lipid deposition. Compared to the Veh group, the expression of the hepatic lipid synthesis regulator SREBP1 significantly rose in both the PS‐L and PS‐H groups (Figure 6A,B). Furthermore, the lipid synthesis‐related protein p‐ACC1^Ser79^ and FASN were markedly increased (Figure 6A,C,D). In addition, FASN enzymatic activity was significantly elevated by 44.1% and 82.5% in the PS‐L and PS‐H groups, respectively, compared with the Veh group (Figure 6E).

In terms of lipid oxidative degradation, the levels of TAG lipase ATGL and the key β‐oxidation enzyme CPT1α significantly declined in both the PS‐L and PS‐H groups (Figure 6A,F,H), while ACOX1 exhibited a significant decrease only in the PS‐H group (Figure 6A,G). Regarding lipid transport, ApoB expression was diminished in both PS‐L and PS‐H groups compared to the Veh group (Figure 6A,I).

We also measured hepatic toxicity markers in mouse serum, such as alanine transaminase (ALT) and aspartate transaminase (AST), but found no significant differences among the groups (data not shown). However, we observed that while there were no differences in serum TAG and TC levels between the groups, FFA levels exhibited a dose‐dependent decline (Figure 6J). These results suggested that the hepatic lipid deposition induced by prolonged PS‐MPs exposure may be in the early stage, characterized by excessive hepatic uptake of FFA and accumulation of hepatic TAG.

## 4 Discussion

In the present study, we investigated hepatic lipid metabolism abnormalities in healthy C57BL/6N mice exposed to PS‐MPs through drinking water. Our most remarkable findings include the following: (1) a single oral gavage of F‐PS‐MPs led to their absorption through the gastrointestinal tract, with predominant accumulation observed in the liver and testes; (2) chronic exposure to PS‐MPs resulted in lipid deposition and abnormal polarization of hepatic KCs toward the M1 phenotype in the liver; (3) hepatic lipid accumulation was mediated by increased lipid synthesis, along with suppressed lipid oxidation and export.

Existing studies have primarily investigated MPs sized around 5 μm [15, 28, 29]. However, more than 80% of MPs detected in the gastrointestinal tract and muscle are smaller, with diameters under 3 μm [30]. Consistent with previous results, significant deposits of PS‐MPs were found in the testes of mice after 24 h of gavage with PS‐MPs of two different particle sizes: 4 μm and 10 μm [31]. Our SEM analysis of PS‐MPs revealed smooth, spherical shapes, with no significant differences in particle size. Zeta potential testing confirmed good stability and uniform distribution in water [32], demonstrating that the PS‐MPs were stable and monodispersed, suitable for further analysis. In adults, the estimated ingestion rate of MPs sized 0–1 mm is approximately 0.1 to 5 g per week [33]. For infants, the estimated mean daily exposures to polyethylene glycol terephthalate (PET) and polycarbonate (PC) MPs from their diet are 83 000 ng/kg body weight and 860 ng/kg body weight, respectively, which are significantly higher than the corresponding values for adults (PET: 5800 ng/kg body weight per day; PC: 200 ng/kg body weight per day) [34]. Additionally, studies involving 8‐week‐old male ICR mice have administered a daily MPs intake of around 4.3 × 10^6^ items, equating to approximately 1.8 mg/kg [25]. Our selection of 1 mg/L and 5 mg/L PS‐MPs doses was based on environmental data [15, 25] and human exposure estimates [4], aiming to encompass both realistic exposure scenarios—defined as environmentally relevant concentrations of PS‐MPs based on reported levels in drinking water and food—and elevated exposure scenarios, which reflect concentrations commonly used in toxicological effect modeling. This range allows for a comprehensive assessment of potential health effects associated with varying levels of MPs exposure. We acknowledge the complexity of accurately translating environmental concentrations to human exposure doses and the need for further research to refine these estimates.

Numerous studies have demonstrated that MPs accumulate in the liver via the gastrointestinal tract [20, 35, 36]. Reports indicate that male mice administered PS‐MPs of sizes 0.5 μm, 4 μm, and 10 μm via gavage exhibited significant accumulation in the testes, with the 4 μm and 10 μm particles displaying a higher propensity for accumulation [31]. In the present study, we also observed significant accumulation of PS‐MPs in both the liver and testes following the gavage administration of 0.2 mg of PS‐MPs. The fluorescence intensity peaked at 2 h likely reflects maximal gastrointestinal absorption of PS‐MPs, consistent with previously reported pharmacokinetic profiles of MPs [37]. During PS‐MPs administration, we observed that there was no significant change in body composition in the mice until week 6. However, from the 9th week until the end of the 12‐week period, there was a marked increase in body fat percentage and a significant decrease in lean mass percentage. These changes in body composition were not attributed to alterations in food or water intake. The observed hepatic accumulation further corroborated previous studies indicating that MPs exacerbate liver lipid deposition [25, 38]. In addition to the potential alterations in systemic energy expenditure, several other mechanisms may account for the increased body fat percentage observed under equivalent dietary intake conditions following chronic PS‐MPs exposure. First, MPs may disrupt gut microbiota composition and intestinal integrity, enhancing dietary energy extraction and nutrient absorption, which can promote fat accumulation without increased food consumption [39, 40]. Second, PS‐MPs exposure has been associated with chronic low‐grade inflammation, which is known to facilitate adipose tissue expansion by promoting lipogenesis and inhibiting lipolysis through pro‐inflammatory cytokines [41]. Finally, environmental stressors such as PS‐MPs could alter the behavior and differentiation capacity of adipose progenitor cells, favoring adipogenesis through modulation of transcription factors like PPARγ and C/EBPα [42, 43]. Collectively, these potential mechanisms may contribute to the observed adiposity phenotype independent of dietary intake, and they warrant further investigation in future mechanistic studies.

In this study, we used C57BL/6N mice maintained on a standard chow diet to investigate the direct hepatic effects of chronic PS‐MPs exposure. This design aimed to simulate baseline environmental exposure in the general population while minimizing dietary confounding. Although a high‐fat diet (HFD) model could better reflect metabolic disorders such as obesity and insulin resistance, resource limitations precluded its inclusion in the current work. Recent studies suggest that PS‐MPs may exacerbate metabolic dysfunction under obesogenic conditions. For instance, PS‐MPs have been shown to aggravate hepatic steatosis, adipose tissue inflammation, and glucose metabolic disorders in HFD‐fed mice [41, 42, 43]. Moreover, PS‐MPs exposure has been linked to alterations in lipid metabolism and endoplasmic reticulum stress through activation of PERK‐ATF4 and NF‐κB signaling pathways [28, 44]. We also recognize that metabolic tests such as glucose tolerance test (GTT) and insulin tolerance test (ITT) are valuable tools to evaluate systemic metabolic responses. While these assessments were not performed in this study, we plan to include them in future work to better characterize the metabolic impact of PS‐MPs exposure under both normal and HFD‐fed conditions [25, 45].

Despite observing significant increases in body fat percentage and hepatic lipid accumulation following chronic exposure to PS‐MPs, several important physiological parameters, including fecal output, physical activity, and body temperature, were not assessed in this study. This was primarily due to experimental and technical constraints that limited our ability to perform metabolic cage analyses. Additionally, while fecal analysis could provide valuable insights into the excretion and potential accumulation of PS‐MPs, compositional analysis of MPs in fecal samples was not performed. These limitations may restrict a more comprehensive understanding of the systemic metabolic alterations induced by PS‐MPs exposure. For example, changes in energy expenditure, thermogenesis, and gut motility could all influence the observed phenotypes. Future studies incorporating metabolic cage measurements, behavioral tracking systems, and precise quantification of MPs in excreta will be essential to further elucidate the physiological and metabolic impact of chronic microplastic exposure in vivo.

Previous studies suggest that PS‐MPs can disrupt lipid metabolism through both direct and indirect mechanisms. Direct effects have been demonstrated in liver organoids, where PS‐MPs induced hepatotoxicity and disrupted lipid metabolism [46]. Additionally, PS‐MPs were shown to impair lipid metabolism by activating the PERK‐ATF4 signaling pathway, leading to increased lipid synthesis [44]. PS‐MPs exposure has also been linked to altered adipose tissue function, suggesting direct interference with lipid storage and metabolism [47]. Conversely, PS‐MPs can indirectly affect lipid metabolism by triggering inflammatory responses. High concentrations of PS‐MPs have been reported to induce intestinal inflammation via natural killer cells, disrupt gut microbiota, and modulate nutrient metabolism [48]. Furthermore, PS‐MPs exposure led to liver tissue damage and abnormal lipid metabolism accompanied by inflammation in diabetic mice [45], while long‐term exposure caused lipid metabolic disorders and cardiovascular damage in rats [49]. Previous studies suggest that inflammatory activation of KCs is often associated with lipid uptake and morphological changes. Under pathological conditions such as nonalcoholic fatty liver disease (NAFLD), KCs can internalize excess lipids, leading to intracellular lipid droplet accumulation and a shift toward a pro‐inflammatory phenotype [50, 51]. This lipid overload not only promotes the secretion of inflammatory cytokines but also contributes to hepatic injury and fibrogenesis, highlighting the dual role of KCs in lipid handling and immune regulation during liver steatosis [52]. These findings indicate that PS‐MPs may impair lipid metabolism through both direct cellular effects and indirect inflammation‐mediated pathways. Further studies are needed to delineate these mechanisms.

KCs, the resident macrophages of the liver, serve as primary phagocytes that efficiently clear PS‐MPs [53]. In the liver, KCs can polarize into two distinct subtypes: M1 KCs, which are linked to pro‐inflammatory responses and the production of pro‐inflammatory cytokines, and M2 KCs, which are associated with anti‐inflammatory responses [54]. In a study where male mice were exposed to 0.5 mg/100 μL of 0.5 μm PS‐MPs in drinking water for 28 days, the proportion of M1 KCs in the liver significantly increased, while the proportion of M2 KCs markedly decreased [55]. Our findings indicated that exposure to 1 μm PS‐MPs also significantly elevated the proportion of M1 KCs while reducing the proportion of M2 KCs in the liver. This effect became more pronounced with increasing doses of PS‐MPs, suggesting a dose‐dependent induction of liver inflammation by PS‐MPs.

M1 KCs synthesize and release various pro‐inflammatory cytokines, including IL‐1, IL‐6, and IL‐17, which amplify inflammation and attract additional immune cells [22]. The persistent pro‐inflammatory phenotype of M1 KCs is associated with the transition from acute to chronic inflammation and subsequent tissue damage [56]. Conversely, M2 KCs secrete several anti‐inflammatory mediators, such as TGF‐β, IL‐4, and IL‐10 [57]. Previous studies suggest that inflammatory activation of M1 KCs is often associated with lipid uptake and morphological changes [50, 51, 52]. In our study, we observed exposure to PS‐MPs significantly increased the expression of the pro‐inflammatory cytokine *Il17a* in the liver of mice, while the levels of the anti‐inflammatory cytokines *Il10* and *Tgfb1* significantly decreased. These findings suggested that chronic exposure to PS‐MPs may trigger a liver inflammatory response due to an imbalance in KCs polarization.

Numerous studies have demonstrated that IL‐17 mediates its inflammatory effects by inducing the expression of chemokines and cytokines through the activation of the NF‐κB signaling [58, 59]. In this study, we observed a significant increase in IL‐17 gene expression, accompanied by elevated levels of p‐IκBα and p‐NF‐κB^p65^. These findings indicated that chronic exposure to PS‐MPs activated the IL‐17/NF‐κB signaling, thereby contributing to chronic liver inflammation. Studies have established a close link between inflammation, immune responses, and the development of metabolic disorders. Notably, NF‐κB not only plays a pivotal role in inflammatory processes but also promotes lipid accumulation in hepatocytes during hepatic steatosis [60, 61]. Furthermore, SREBP1 has been identified as a downstream target of NF‐κB [62], primarily regulating genes involved in the biosynthesis of fatty acids and TAGs in key lipid‐producing organs, including the liver [63]. In this study, we demonstrated that NF‐κB enhances the expression of SREBP1 in the liver. Additionally, we noted a significant increase in the level of p‐ACC1, a key regulator of lipid synthesis, and in FASN activity, while the expression levels of ATGL, ACOX1, and CPT1α—proteins associated with lipid oxidation and degradation—were significantly decreased. The expression of ApoB, which is essential for lipid transport, was also notably diminished. Although APOB is critical for VLDL secretion, reduced hepatic APOB levels can occur alongside triglyceride accumulation due to impaired lipidation or ER stress [64]. Similar observations were reported in PS‐MPs exposure models, indicating disrupted lipid export [65]. This may explain the hepatic lipid retention seen in our study. The decrease in adipose tissue mass may result from enhanced lipolysis during the fasting period before sacrifice. PS‐MPs exposure may further promote fat mobilization by disrupting energy metabolism [28, 66]. Collectively, these results indicate that exposure to PS‐MPs induces liver inflammation, leading to dysregulation of liver lipid metabolism and ultimately resulting in lipid deposition.

While our use of a mouse model with PS‐MPs exposure via drinking water provides a controlled and practical approach to simulate human exposure, it is important to acknowledge potential limitations regarding the generalizability of the findings to humans. Animal models, while valuable, may not fully replicate the complex physiological, metabolic, and environmental factors that influence human responses to PS‐MPs exposure. The single exposure route (drinking water) used in this study, though relevant, does not capture other potential exposure pathways such as inhalation, dermal contact, or dietary intake, which may contribute to PS‐MPs exposure in real‐world scenarios. This limitation underscored the need for future studies incorporating multiple exposure routes for a more comprehensive understanding. Furthermore, the modest sample size (*n* = 6–7 per group) may limit the statistical power to detect subtle effects, and potential inter‐individual variability might not have been fully captured. Additionally, residual unrecognized confounding factors inherent in experimental designs, such as variations in drinking water consumption or physiological differences among the animals, may have influenced the outcomes. Future investigations addressing these limitations with larger sample sizes, diverse exposure routes, and complementary human epidemiological data would enhance the generalizability and translational relevance of findings related to PS‐MPs exposure.

## 5 Conclusion

In this study, we found that short‐term exposure to PS‐MPs resulted in the liver and testes being identified as the primary accumulation depots. Long‐term exposure to PS‐MPs led to an increase in body fat composition and hepatic lipid deposition. These changes were mediated by abnormal polarization of KCs in the liver, which induces chronic inflammation through the IL‐17/NF‐κB signaling pathway, resulting in dysregulation of hepatic lipid metabolism. These findings underscore the potential health risks associated with waterborne PS‐MPs exposure, highlighting the need for further research on the implications of MPs pollution on lipid metabolic disorders.

## Author Contributions

T.L., Z.Y., Y.H., R.L., L.Z., and W.G. conducted experiments and generated data. R.C. was involved in collecting and analyzing data. W.G. was responsible for manuscript editing. W.G. designed the experiments. Z.Y. drafted the manuscript. The accuracy of the data analysis and the integrity of the data were fully guaranteed by W.G., who were also the guarantor of this work and had complete access to all the data. The final manuscript was read and approved by all contributors.

## Conflicts of Interest

The authors declare no conflicts of interest.

## Supporting information


**Figure S1:** Body weight (A), average food intake (B), and average water intake were monitored during PS‐MPs exposure. Data are presented as mean 00B1; SD (*n* = 7).
**Figure S2:** Representative Oil Red O staining of liver sections from Veh and PS‐MPs groups after exposure. Scale bar = 50 μm.

## Data Availability

The datasets used and/or analyzed during the current study are available from the corresponding author on reasonable request.
