# Supplementary material for: Chronic Waterborne Exposure to Polystyrene Microplastics Induces Kupffer Cell Polarization Imbalance and Hepatic Lipid Accumulation
Source: FASEB J. 2025 Sep 3;39(17):e70980. doi: 10.1096/fj.202500910RR (PMC12406979; doi:10.1096/fj.202500910RR)
Supplement: Supplementary file 1 — Figure S1: Body weight (A), average food intake (B), and average water intake were monitored during PS‐MPs exposure. Data are presented as mean 00B1; SD (n = 7). Figure S2: Representative Oil Red O staining of liver sections from Veh and PS‐MPs groups after exposure. Scale bar = 50 μm. [file FSB2-39-e70980-s001.docx]

**Captions of Supplementary Figures**

**
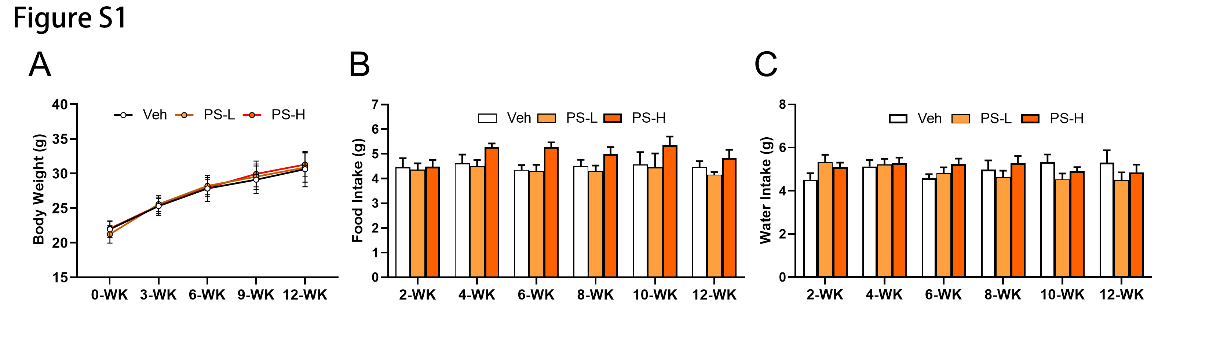
**

**Figure S1.** Body weight (A), average food intake (B), and average water intake were monitored during PS-MPs exposure. Data are presented as mean ± SD (n = 7).

**
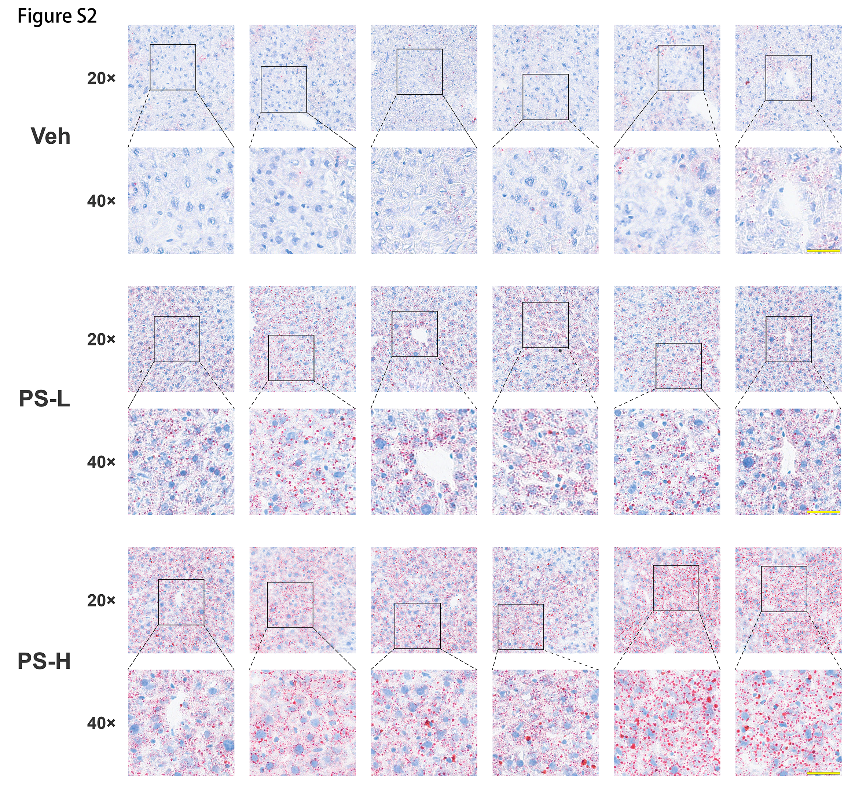
**

**Figure S2.** Representative Oil Red O staining of liver sections from Veh and PS-MPs groups after exposure. Scale bar = 50 μm.
